# Archetypal type II and III *Toxoplasma gondii* oocysts induce different immune responses and clinical outcomes in experimentally infected piglets

**DOI:** 10.3389/fimmu.2022.1021556

**Published:** 2022-10-20

**Authors:** Andrea Largo-de la Torre, Carlos Diezma-Díaz, Rafael Calero-Bernal, Gabriela Atencia-Cibreiro, Roberto Sánchez-Sánchez, Ignacio Ferre, Javier Regidor-Cerrillo, Luis Miguel Ortega-Mora

**Affiliations:** ^1^ SALUVET-Innova, Faculty of Veterinary Sciences, Complutense University of Madrid, Madrid, Spain; ^2^ SALUVET, Animal Health Department, Faculty of Veterinary Sciences, Complutense University of Madrid, Madrid, Spain

**Keywords:** *Toxoplasma gondii*, piglets, oocysts, type II and type III isolates, immune response, clinical outcome, parasite dissemination

## Abstract

Livestock animals, such as swine, are an important source of *Toxoplasma gondii* in the human population. Currently, there is limited knowledge regarding the potential influence that the *T. gondii* genotype might exert on establishing infection in swine. Herein, we investigated the role of 2 *T. gondii* isolates, type II and III, representative of the genotypes circulating in Europe, in the immune responses and infection dynamics in piglets. Recently obtained oocysts (10^3^) from the *T. gondii* field isolates TgShSp1 (type II, ToxoDB genotype #3) and TgShSp24 (type III, #2) were used for oral infection. Thirteen 50-day-old female piglets of the Landrace-Large White crossbreed were randomly allocated into three different groups: Group 1 (G1, n=5), inoculated with TgShSp1; Group 2 (G2, n=5), inoculated with TgShSp24; and Group 3 (G3, n=3), a non-infected control group. Clinical signs were monitored daily until 42 days post-infection (dpi) when piglets were euthanized. Blood samples were collected weekly to test the cellular immune response in parasite-stimulated peripheral blood and specific IgG, IgG1 and IgG2, responses in sera. Parasite distribution and burden were evaluated in target tissues using a mouse bioassay and quantitative RT−PCR (qPCR). Apathy and a moderate decrease in feed consumption were observed in G1 and G2 piglets between 5 and 8 dpi, coinciding with fever (>40°C). G2 piglets had higher temperatures for a longer duration. Using mouse bioassay and qPCR, the detection frequency was higher in G2 *vs.* G1, and the highest parasite burdens in target tissues were also found in G2. Seroconversion was detected at 14 dpi in both infected groups, but higher antibody levels were observed in G2 piglets. Cytokine analyses revealed the production of IL-8, IL-1β and IFN-ɤ from 7 dpi in both infected groups. Moreover, IL-12 was produced from 7 dpi in G1 and from 14 dpi in G2. Levels of IL-8 were higher in G2, but IL-1β, IL-12 and IFN-ɤ were higher in G1 at 14 dpi. This cytokine profile reveals a predominant proinflammatory response that could be involved in limiting *T. gondii* infection in piglets, although it is more efficient against TgShSp1 type II-driven infection.

## Introduction

*Toxoplasma gondii (T. gondii)*, one of the most prevalent protists worldwide, is an apicomplexan parasite able to chronically infect all warm-blooded animals, including humans. As a facultative heteroxenous parasite, it has several potential routes of transmission within and between different host species, including three invasive stages: i) tachyzoite, the rapidly replicating form; ii) bradyzoites, the slowly replicating or quiescent form harboured in tissue cysts; and iii) sporulated oocysts bearing sporozoites (1). Main routes of infection include horizontal transmission by oral ingestion of infectious oocysts shed in the faeces of specific feline definitive hosts or of tissue cysts contained in raw or undercooked meat from infected intermediate hosts and vertical (transplacental) transmission of tachyzoites, and both transmission routes are contributing to the complex epidemiology of *T. gondii* infection (1, 2). Most infected humans are asymptomatic or have mild clinical signs; nevertheless, *T. gondii* can cause severe neurologic damage and even death of the foetus when acquired during pregnancy in humans and other mammals. It can also cause severe disease, including encephalitis and pneumonia, in immune-compromised patients, which may also result in death (3).

Pigs are intermediate hosts that usually bear *T. gondii* tissue cysts in their organs and muscles and therefore are prone to constitute one of the primary sources of human infection. Serological surveys have shown that up to 30% of domestic pigs have been exposed to *T. gondii* worldwide, with a global seroprevalence of approximately 19% (4). Viable *T. gondii* has been frequently isolated using bioassays in mice from pork muscle tissues obtained at slaughterhouses [reviewed in (4)]. A few studies dealt with the detection of parasite DNA in pig tissues using different PCR assays, and the most reliable data indicated an average prevalence of approximately 21% (2-66%) in China, and 31% (5-57%) in European Union (EU) countries (Italy, Poland, Czech Republic) (4). Likewise, the European Food Safety Authority (EFSA) recognizes *T. gondii* as a public health hazard in swine that should be assessed during meat production (5).

Similar to humans, *T. gondii* infection is usually asymptomatic in pigs. As observed in previous studies, some animals may develop mild clinical signs, such as fever, apathy or lack of appetite, during the acute phase of the disease and fully recovering during the chronic phase (4, 6–9). Although clinical toxoplasmosis in swine is rare, outbreaks of toxoplasmosis in pigs have been reported in China and Brazil (10, 11). In addition, although *T. gondii* may cause reproductive failure in pigs, this is a rare finding compared to losses in other species, such as small ruminants (2). In this regard, pioneer experimental infections were able to link lesions (e.g., necrotizing placentitis, nonsuppurative encephalomyelitis and myocardial degeneration, necrosis and mineralization) to congenital toxoplasmosis (12). Most recently, a Swiss study reported natural placental infection by *T. gondii* in at least 3.5% of sows with reproductive disorders and immunohistochemically evidenced the infection in the placenta of one sow (13). Nevertheless, mild clinical signs are accompanied by wide parasite dissemination in pig target tissues (brain, heart and muscle with commercial interest), as described after oocyst or tissue cyst experimental infections in which viable *T. gondii* was maintained for the chronic phase of infection after four to six months post-infection in these tissues (4).

The population structure of *T. gondii* is predominantly clonal in the Northern Hemisphere. Regarding clonal types that circulate in European livestock, several genotyping-based studies have demonstrated the predominance of type II strains, which coexist with lower percentages of type III and recombinant strains (14). Previous studies in rodents highlighted marked differences in virulence depending on the clonal type, primarily related to specific mechanisms to subvert host proinflammatory immune responses (15–17). Recent reports described a wide range of virulence in the outbred mouse model caused by isolates belonging to types II and III, in which type III isolates induced the highest mortality rate and cumulative morbidity values compared to isolates of type II (18, 19). However, virulence in mice is not necessarily representative of what occurs in other relevant hosts, such as sheep (20). There is currently limited knowledge regarding the influence of genotype on establishing infection and disease outcome in swine. Interestingly, acute clinical toxoplasmosis described in Brazil and China was related to the *Toxoplasma* clonal type Chinese 1; animals had a history of apathy, dyspnoea, anorexia, and weight loss and died 4-5 days after first showing clinical signs (10, 11). Because of the scarce literature available (2), comparative studies using different *T. gondii* genotypes are still necessary to investigate the influence of the isolate on parasite dissemination, immune response and disease presentation and outcome. Previous studies based on experimental infection of piglets with a hybrid type I/II strain compared to a type II strain revealed differences in parasite burden (8) with earlier antibody production and control in animals infected with the type II strain (21). The present study aimed to characterize the influence of two archetypal type II and type III isolates of *T. gondii*, TgShSp1 (Type II, genotype#3) and TgShSp24 (Type III, #2), on the clinical outcome and infection dynamics in piglets. Evaluation of virulence in mice classified both *T. gondii* isolates as “non-virulent” (< 30% cumulative mortality), but the TgShSp24 isolate exhibited higher degrees of virulence (19). This study defines the host–parasite interactions for these isolates during infection in piglets.

## Material and methods

### Piglets

Thirteen female Landrace-Large White crossbreed piglets 50 days old and weighing approximately 25-28 kg from a high sanitary status farm (Agropardal de Almendros S.L., Cuenca, Spain) were used for the experimental infection. The absence of specific antibodies against *T. gondii* and against porcine reproductive and respiratory syndrome virus in the recruited piglets was confirmed by PrioCHECK™ Porcine Toxoplasma Ab Kit (ThermoFisher Scientific, Massachusetts, MA, USA) and IDEXX PRRS X3 Ab Test (IDEXX, Westbrook, ME, USA) commercial ELISA tests, respectively, following the manufacturer’s instructions.

### Parasites and inoculum

Recently obtained oocysts from two different *T. gondii* isolates, TgShSp1 (Type II, ToxoDB genotype#3) and TgShSp24 (Type III, #2), were used as inocula (20, 22). Details on the isolates are provided in **Table 1**. Oocysts were obtained through oral infection of cats as previously described (20). Briefly, Swiss/CD1 mice were intraperitoneally inoculated with cell culture-derived tachyzoites of both isolates that had been kept at low cell culture passage (n<10), and 2 months post-inoculation, mice were euthanized, and the brains were collected to feed the kittens. To prepare the inocula, sporulated oocysts were quantified using a single-use Neubauer chamber (DHC-N01 Neubauer Improved CYTO, Gentaur, UK) and subsequently diluted in PBS to a concentration of 10^3^ oocysts per mL. The oocysts used for infections were maintained at 4°C for 1.5 years from production until inoculum preparation. Oocyst infectivity was previously evaluated in an established mouse model of infection as described (20) (data not shown).

**Table 1:** *Toxoplasma gondii* isolates used in the present study.

Isolate	Genotype (ToxoDB#)	Clonal type	Host of origin	Mouse virulence (cumulative mortality, %)*
TgShSp1	#3	Type II	Ovine foetal brain	non-virulent (0)
TgShSp24	#2	Type III	Adult ovine myocardium	non-virulent (18.2)

*Strains detailed description is reported in (19).

*Toxoplasma gondii* tachyzoites for the in-house ELISAs, the peripheral blood stimulation assay and the standard curve for quantitative real-time PCR (qPCR) were obtained from the Tg ME49 isolate (the reference type II isolate), which was maintained *in vitro* by continuous passage in VERO-81 cell cultures using DMEM supplemented with 1% foetal bovine serum. Tachyzoites were purified, filtered by Cyclopore^®^ polycarbonate membranes (10 µm) (Whatman^®^, Buckinghamshire, UK) and pelleted by centrifugation at 1350×g for 10 minutes at 4°C. Pellets containing 1x10^8^ tachyzoites per vial in 4 ml of PBS were stored at -80°C until lyophilization as previously described (23). On the other hand, pellets containing different amounts of tachyzoites were maintained at -80°C until use for the qPCR standard curve.

### Experimental design and clinical monitoring

**Figure 1:**
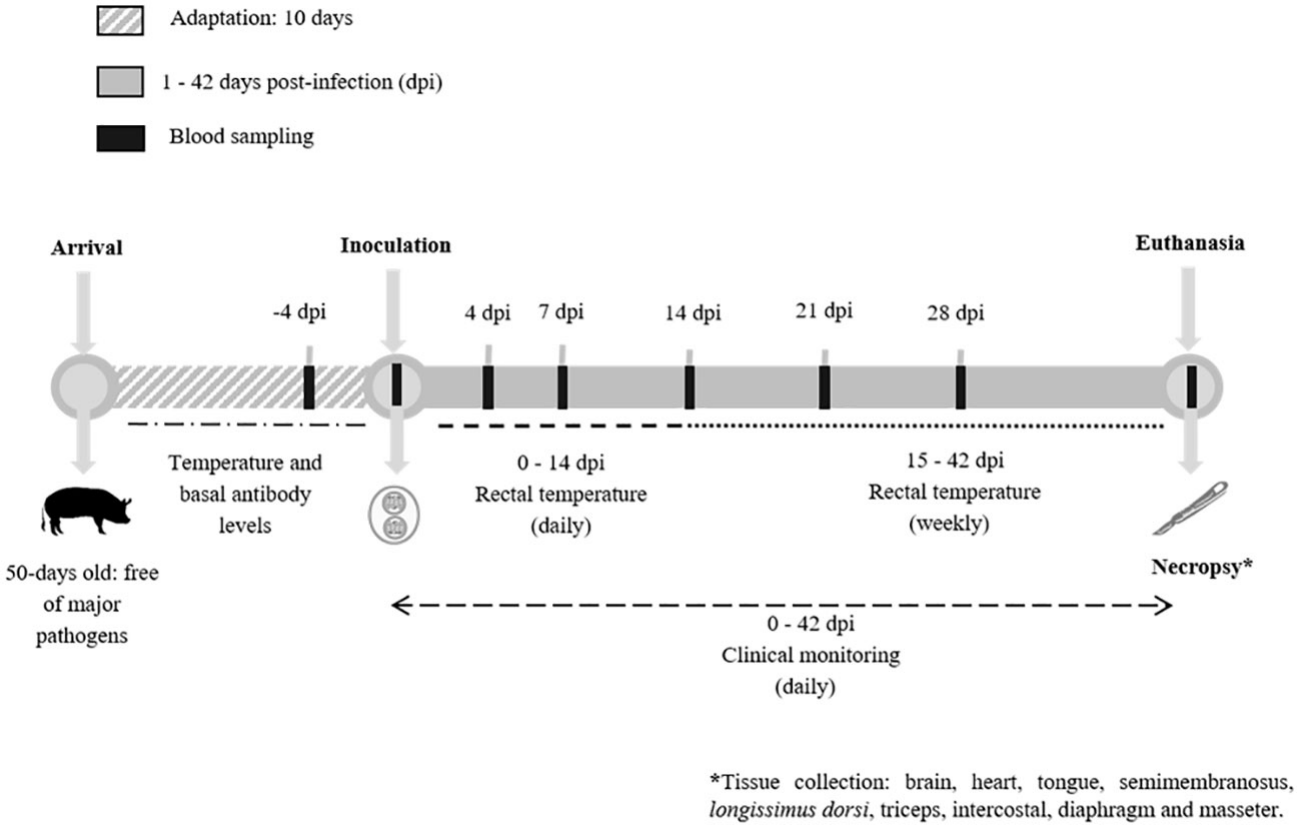
Experimental design of *Toxoplasma gondii* infections in piglets. Timeline, clinical monitoring and sampling throughout the experimental infection of piglets with *T. gondii* oocysts.

After an adaptation period of 10 days, piglets were randomly allocated into two groups of five animals each (G1 and G2) and one group of three individuals (G3); each group was independently housed in conditioned boxes with controlled room temperature and humidity, bedding, and *ad libitum* water and food throughout the trial (**Figure 1**).

Piglets were orally dosed with 10^3^ oocysts of TgShSp1 –Type II- (G1) or TgShSp24 –Type III– (G2) in 1 mL of PBS by direct deposition on the piglets’ oral cavity using a syringe. Group 3 (G3) was orally inoculated with PBS and served as the non-infected control group. All necessary control measures were taken to avoid cross contamination with oocysts between boxes, such as changing shoe covers and clothes, and avoiding shared material between boxes. All waste material was disposed of by autoclaving and incineration.

Clinical signs were monitored daily throughout the entire experimental period by two veterinarians, focusing on the possible detection of diarrhoea, anorexia, apathy and behavioural changes. Rectal temperatures were recorded daily from 4 days prior to inoculation (considered the mean basal temperature) to 14 days post-infection (dpi) and then weekly until the end of the experiment (42 dpi). Any temperature above 40°C was considered as fever. At the end of the experiment, the animals were sedated by intramuscular administration in the neck with a cocktail containing ketamine (5 mg/kg) (Anesketin, Dechra, Northwich, UK), xylazine (5 mg/kg) (Xilagesic, Calier, Les Franqueses del Vallés, Barcelona, Spain), butorphanol (0,2 mg/kg) (Butomidor, Richter Pharma, Wels, Austria) and tiletamine with zolazepam (2 mg/kg) (Zoletil, Virbac, Carros, France), and then immediately euthanized by an intravenous overdose of pentobarbital (Dolethal, Vetoquinol, Nymburk, Czech Republic).

### Sampling

Blood samples were collected for immune response studies at -4, 0, 4, 7, 14, 21, 28 and 42 dpi by cranial cava venipuncture using vacuum devices with and without heparin anticoagulant (BD Vacutainer^®^ Plus Plastic Serum, Franklin Lakes, NJ, USA) coupled to 21-18G needles (Terumo™, ThermoFisher Scientific, MA, USA). After extraction, blood samples collected without anticoagulant were allowed to clot and were centrifuged (1200×g, 10 minutes, 4°C) to obtain serum samples. Sera were stored at -20°C until serological tests. Blood samples collected with anticoagulant were used for the blood immunostimulation assay (see *Peripheral blood stimulation assay*) and subsequent study of the cellular immune responses by measuring cytokine production after specific stimulation in cell supernatants.

During necropsy, approximately 50 g of tissue samples were collected from brain, diaphragm, heart, tongue, and skeletal muscles, including masseter, intercostal, *longissimus dorsi*, *triceps brachii* (triceps) and semimembranosus, for parasite detection by mouse bioassay and/or quantification by qPCR. Tissue samples were stored at 4°C until acid-pepsin artificial digestion (see *Tissue digestion and mouse bioassay*).

### Tissue digestion and mouse bioassay

Tissue homogenates were obtained by acid-pepsin artificial digestion as previously described (6). In addition, brain homogenates were obtained as previously described without acid-pepsin treatment (24). Briefly, brain tissues were homogenized in PBS, filtered through sterile gauze and then centrifuged at 1350×g for 15 minutes at 4°C.

Six-week-old female Swiss/CD1 mice (Janvier Labs, Le Genest-Saint-Isle, France) were used for the bioassay. Mice were housed in a controlled environment with 12-h light and 12-h dark cycles and provided rodent feed and water *ad libitum*. Brain sediment and acid-pepsin-treated homogenates from the heart, tongue, *longissimus dorsi*, triceps and semimembranosus were used for mouse inoculation. Specifically, three mice per tissue were subcutaneously injected with 500 μL of each homogenate (100 mg of extract per mouse) diluted 1:3 in PBS with 1% antibiotic solution (penicillin (1000 IU/ml; Sigma−Aldrich, Madrid, Spain) and streptomycin (100 μg/ml; Sigma−Aldrich). Animals were examined at least twice a day for clinical signs compatible with toxoplasmosis. Mice that displayed severe loss of body condition, severe respiratory distress or nervous clinical signs were humanely euthanized. All remaining mice were euthanized at 30 dpi. During necropsy, brain (for qPCR) and serum samples were collected and stored at −20°C until IgG and IgM serological analyses. Animals positive by qPCR and/or serology were considered infected.

### DNA extraction and quantitative real-time PCR (qPCR) for parasite detection and quantification

Genomic DNA was extracted in triplicate from 50 µL of each tissue digest (see *Tissue digestion and mouse bioassay*) obtained from piglets and from 50 to 80 mg from the brains of mice of the bioassay (see *Tissue digestion and mouse bioassay*) using a commercial DNA purification kit (Maxwell RSC Tissue DNA kit, Promega, Wisconsin, USA) following the manufacturer’s instructions.

Detection in the mouse brain from the bioassay was performed using 529 bp repetitive element (RE) template-based qPCR under PCR conditions as previously described (25). Detection and quantification of parasite DNA on target tissues from piglets was performed as described above with slight modifications in cycling parameters: preheating at 95°C for 10 min and then 40 two-step cycles of 95°C for 15 s and 65°C for 30 s. The standard curve was constructed using 10-fold serial dilutions of known copy numbers of the *T. gondii* tachyzoites (see *Parasites and inoculum*) and diluted in pig DNA (20 ng/µl). The slope (S between -3.5 and -3.2) and regression coefficient (R^2^ > 0.991) of the standard curves were also calculated to estimate the efficiency of the assay.

### Peripheral blood stimulation assay

Heparinized blood samples were processed within 2 h of collection by mixing 500 μL blood with 500 μL RPMI 1640 medium (Gibco, Paisley, UK) supplemented with 10% foetal bovine serum (FBS; Thermo Fisher Scientific, Waltham, USA) and 1% antibiotic/antimycotic solution (Lonza, Basel, Switzerland). Blood cells were cultured in 24-well plates (Thermo Fisher Scientific, Waltham, USA) in the presence of 10^6^ lyophilized *T. gondii* tachyzoites (see *Parasites and inoculum*) as antigen, concanavalin A as a control (ConA, Sigma−Aldrich, Madrid, Spain), both at final concentrations of 5 μg/mL, or PBS as a negative control. The plates were incubated in a 5% CO_2_/37°C/100% humidity atmosphere for 48 h and centrifuged at 1000×g for 10 min at 4°C. Finally, cell-free culture supernatants were stored at −80°C until analysis.

### Humoral immune responses

*Toxoplasma gondii*-specific IgG was identified in piglets using a widely used commercial indirect ELISA test according to the manufacturer’s instructions (PrioCHECK™ Porcine Toxoplasma Ab Kit, ThermoFisher Scientific, MA, USA). This probe was provided with plates coated with formalin-fixed cell culture-derived tachyzoite antigen. Serum samples were used at a 1:50 dilution, and the colorimetric reaction was measured at a wavelength of 450 nanometres (nm). The results were normalized by calculating the percent positivity value (PP) in relation to the optical density (OD) values for each sample by applying the following formula: PP sample = (OD_450_ sample/OD_450_ positive control) x 100, according to the manufacturer’s indications. Samples with PP values greater than or equal to 20 were considered positive, while samples with PP values below 20 were considered negative.

Moreover, an in-house ELISA using whole lyophilized TgME49 tachyzoites as antigen (see *Parasites and inoculum*) (23) was used to determine IgG, IgG1 and IgG2 levels in piglets and IgG and IgM levels in mice. Briefly, 96-well microtiter plates (Thermo Scientific, Fisher Brand Maxisorp^®^, MA, USA) were coated with 10^5^ tachyzoites in 50 mM sodium carbonate buffer (pH 9.6) and blocked in 5% powdered skim milk in 0.05% PBS-Tween. Sera samples were diluted 1:100 in blocking solution and after washing, were incubated with secondary antibodies.

For piglet sera, the secondary antibody was protein G + HRP (1:6000; P8170-250U, Sigma Aldrich, Madrid, Spain), and the bound antibodies were detected by incubation with TMB ultra (34028, Thermo Fisher Scientific, Waltham, USA) in the dark. After 10 min, the reaction was stopped by adding sulfuric acid, and the absorbance was measured as optical density values at 450 nm. Piglet sera samples were also analysed by ELISA to determine antibody values and IgG isotype ratio (IgG1/IgG2) as described above using anti-porcine mouse IgG1 (MCA635GA) and IgG2 (MCA636GA) monoclonal antibodies (BIO-RAD, California, USA) at 1:2500 (IgG1) or 1:6000 (IgG2) dilutions, followed by anti-mouse IgG – peroxidase antibody (1:10000 for IgG1 and 1:5000 for IgG2) produced in rabbit (Sigma Aldrich, Missouri, USA).

For mouse sera, the secondary antibodies were anti-mouse peroxidase-conjugated IgG (1:10000; A9044, Sigma Aldrich, Madrid, Spain) and IgM (1:500; AP128P, Sigma Aldrich, Madrid, Spain). The bound antibodies were detected by incubation with ABTS (Roche, Basel, Switzerland) in the dark for 20 min. The reaction was stopped by adding oxalic acid, and absorbance was measured as the optical density value at 405 nm.

OD values were determined using a relative index percent (RIPC) employing the following formula: RIPC = (OD_λ_ sample – OD_λ_ negative control)/(OD_λ_ positive control – OD_λ_ negative control) x 100. Samples were considered positive (i.e., animals had seroconverted) when statistically significant differences were observed compared to the negative control.

### Cellular immune responses

Cytokine level quantification (GM-CSF, IFN-γ, IL-1β, IL-4, IL-6, IL-8, IL-10, IL-12, TGF-β1, and TNF-α) was assessed in cell-free culture supernatants using a commercial array-based multiplex ELISA kit (Porcine cytokine array Q1, RayBiotech, Norcross, GA, USA) following the manufacturer´s recommendations. Cytokine concentrations were determined in pooled supernatants for each group throughout the study on 0, 4, 7, 14, 21, 28 and 42 dpi. Pools were made by mixing the same amount of supernatant from each of the animals in the group, and a 1:2 dilution was used for the test. The serum incubation was performed overnight at 4°C with gentle rocking, and biotinylated antibody cocktail and Cy3 equivalent dye-streptavidin incubation were performed at room temperature for 2 h and 1 h, respectively. Fluorescence was measured using a GenePix 4000B laser scanner (580 PMT) in the Genomics Unit, Research Support Centre, Complutense University of Madrid, Spain.

In addition, the two time-points from the experiment showing the highest response or most different values in the pool were selected to study individual responses from specific animals under the same conditions as described above.

IFN-γ production in the supernatants was also individually investigated throughout the experiment using a commercial porcine enzyme immunoassay kit (Porcine IFN-γ ELISA BASIC kit, Mabtech AB, Sweden) following the manufacturer’s recommendations. The colorimetric reaction was developed by the addition of 3,3´,5,5´ tetramethylbenzidine substrate (TMB) (Sigma−Aldrich, Madrid, Spain) following incubation for 5-10 min in the dark. Reactions were stopped by adding 0.2 M H_2_SO_4,_ and the plates were read at 450 nm. Cytokine concentrations were calculated by interpolation from a standard curve generated using recombinant cytokines provided with the probes. Concentration values were only considered when inside the detection limits indicated for each probe by the manufacturer.

### Statistical analysis

Rectal temperatures, serology and IFN-ɤ levels were analysed using a two-way ANOVA repeated measures test followed by a Tukey post-test. Fisher’s exact test was applied to analyse the frequencies of parasite detection in tissues in the mouse bioassay and PCR analysis. Mortality rates in the mouse bioassay were analysed using the Mantel−Cox log-rank test to compare the resulting survival curves. Parasite burden and individual cytokine levels assessed using the array were analysed using the nonparametric Kruskal−Wallis test followed by Dunn’s test for comparisons between groups or the Mann−Whitney test. Statistically significant differences were considered at p < 0.05. GraphPad Prism 6 v.6.01 software (San Diego, CA, USA) was used to perform all statistical analyses and to create all graphical illustrations.

## Results

### Clinical outcome exhibited an elevated fever in piglets infected with the type III TgShSp24 isolate

**Figure 2:**
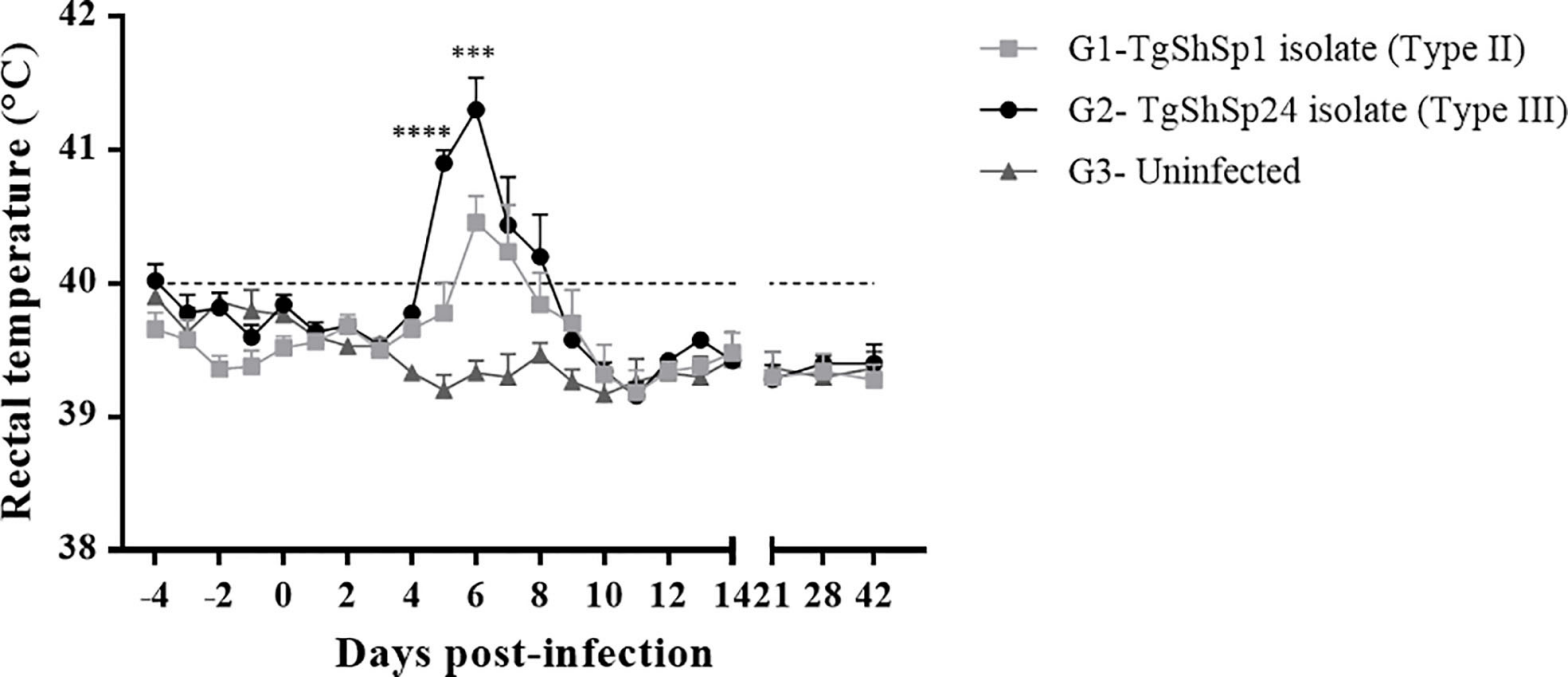
Mean rectal temperatures recorded throughout the study. Rectal temperatures of piglets infected with oocysts from TgShSp1 (G1) and TgShSp24 (G2) isolates and uninfected control (G3) animals. Each point represents the mean value at the different sampling times for each group. Error bars represent the SEM. The horizontal dashed line indicates 40°C. Fever was established at values ≥40°C. Significant differences between the infected G1 and G2 groups are indicated with asterisks: *** indicates p < 0.001 and **** indicates p < 0.0001.

Mean rectal temperatures were recorded daily until 14 dpi and weekly thereafter (**Figure 2**). Basal temperatures in the three groups ranged between 39.7°C and 39.9°C. A significant increase in rectal temperature was detected in all infected animals from G1 between 5 and 7 dpi (p < 0.05; two-way repeated-measures ANOVA, Tukey’s post-test), showing fever (>40°C). All G2 animals extended the period with fever from 5 to 8 dpi (p < 0.05; two-way repeated-measures ANOVA, Tukey’s post-test). In both groups, the temperature peak was recorded at 6 dpi (mean temperature G1 = 40.5°C and G2 = 41.3°C), while all infected groups returned to basal temperature levels at 10 dpi and maintained basal levels until the end of the experiment. A direct comparison between the two infected groups showed that G2 animals presented the highest records of temperature at 5 and 6 dpi (p < 0.001 two-way ANOVA repeated-measures, Tukey’s post-test).

Apathy and a moderate decrease in feed consumption appeared simultaneously with the fever. No diarrhoea or other remarkable clinical signs were observed in any of the piglets throughout the study.

### Dissemination and parasite burden were higher with the type III TgShSp24 isolate

*Toxoplasma gondii* infection was confirmed in all animals and tissues evaluated by mouse bioassay and/or qPCR in the infected groups (G1 and G2). Parasite presence was not detected in tissue samples from the uninfected group (G3).

Using the mouse bioassay, parasite presence was detected in at least one of the target tissues from each animal from G2 (TgShSp24 isolate) and in 4 out of 5 animals (80%) from G1 (TgShSp1 isolate). All mice inoculated with tissues from G1 that were positive by qPCR were also seropositive by ELISA. No antibodies were detected in one animal inoculated with semimembranosus muscle homogenate from G2 that succumbed early to infection at 14 dpi. Mice inoculated with tissue homogenates from G2 exhibited a 23.3% mortality rate at 19 dpi (in decreasing order of percentage from *longissimus dorsi* muscle, heart, brain, tongue and semimembranosus muscle), whereas there was absence of mortality in mice inoculated with tissues from G1 (p <0.01, Mantel−Cox log-rank test). The highest detection frequency by tissue in G1 was found in the heart (66.7%), brain, tongue and semimembranosus muscle (>50%). In G2, the highest frequencies were found in the tongue (100%), heart (86.7%), triceps and semimembranosus muscle (>70%). The tissue with the lowest detection frequency in both groups was the *longissimus dorsi* muscle (13.3% in G1 and 40% in G2) (**Table 2**). No significant differences were detected in the detection frequency between the G1 and G2 groups for any tissue. However, comparison analysis between infected groups G1 and G2, including results from all six analysed tissues, showed that the detection frequency of the parasite was statistically higher in G2 (71.1%) *vs.* G1 (44.4%) (p<0.01, Fisher’s exact test).

**Table 2:** Frequency of *Toxoplasma gondii* detection in tissues of the infected piglets by mouse bioassay and 529pb RE qPCR.

Isolate	Tissue	Bioassay detection (%)/(+/n)	PCR tissue digest detection (%)/(+/n)
TgShSp1 (Type II)	Brain	53.33 (8/15)	60 (9/15)
TgShSp24 (Type III)		46.66 (7/15)	86.66 (13/15)
TgShSp1 (Type II)	Heart	66.66 (10/15)	80 (12/15)
TgShSp24 (Type III)		86.66 (13/15)	100 (15/15)
TgShSp1 (Type II)	Tongue	53.33 (8/15)	66.66 (10/15)
TgShSp24 (Type III)		100 (15/15)	93.33 (14/15)
TgShSp1 (Type II)	Triceps	26.66 (4/15)	46.66 (7/15)
TgShSp24 (Type III)		80 (12/15)	33.33 (5/15)
TgShSp1 (Type II)	Semimembranosus	53.33 (8/15)	33.33 (5/15)
TgShSp24 (Type III)		73.33 (11/15)	60 (9/15)
TgShSp1 (Type II)	*Longissimus dorsi*	13.33 (2/15)	33.33 (5/15)
TgShSp24 (Type III)		40 (6/15)	66.66 (10/15)
TgShSp1 (Type II)	Diaphragm	ND	40 (6/15)
TgShSp24 (Type III)			53.33 (8/15)
TgShSp1 (Type II)	Intercostal	ND	60 (9/15)
TgShSp24 (Type III)			66.66 (10/15)
TgShSp1 (Type II)	Masseter	ND	66.66 (10/15)
TgShSp24 (Type III)			100 (15/15)

ND, not done; +, positive samples; n, total number of samples.

After PCR analyses of the tissue homogenates, the detection frequency was higher than those found by bioassay in almost all tissues (**Table 2**). *Toxoplasma gondii* was detected in at least 5 of 9 tissues selected for analysis in all animals belonging to the infected groups (G1 and G2). Similar to the mouse bioassay, the highest detection frequencies by tissue were found in the heart, tongue and brain in both infected groups (an overall >60% in both challenged groups). In G1 and G2, the detection frequency was > 60% in tissues not assessed by bioassays, such as the intercostal and masseter, except for the diaphragm, which was 40% for G1 and 53.33% for G2 (**Table 2**). Similarly, only the frequency of detection was significantly higher in G2 than in G1 in the masseter muscle (p<0.01, Fisher’s exact test). Nevertheless, considering the detection frequency in all tissues analysed by PCR or uniquely those included in the bioassay for comparison, G2 displayed the highest detection frequency (73.3%) *versus* G1 (54%) (p < 0.01, Fisher’s exact test).

**Figure 3:**
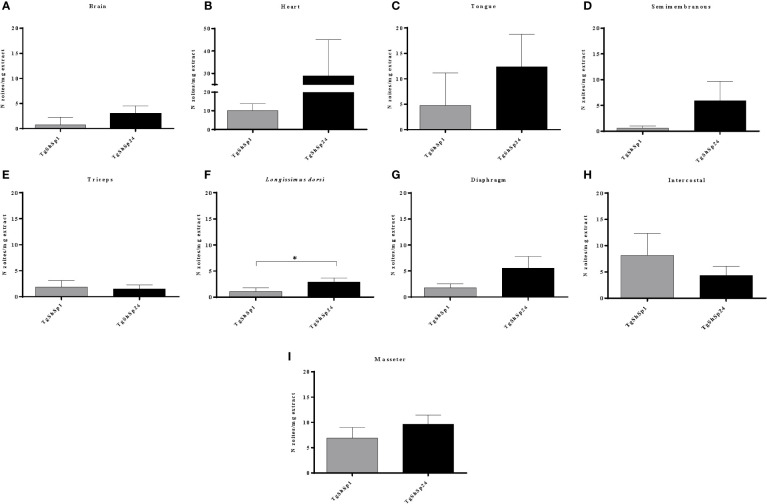
*Toxoplasma gondii* burdens quantified by 529 bp RE qPCR analysis performed on homogenates from pig tissues: brain **(A)**, heart **(B)**, tongue **(C)**, semimembranosus **(D)**, triceps **(E)**, *longissimus dorsi* **(F)**, diaphragm **(G)**, intercostal **(H)** and masseter **(I)**. Column-plot graphs represent the mean parasite burdens as the number of zoites per mg of tissue infected with TgShSp1 and TgShSp24 isolates. Error bars indicate the SEM. *Statistical differences between infected groups (p < 0.05).

The highest parasite burdens in both infected groups G1 and G2 were also observed in the heart (≥ 10 zoite/mg tissue) (**Figure 3B**), followed by the tongue and masseter muscle (≥ 5 < 10 zoite/mg tissue) (**Figures 3C, I**). The brain, which had a high frequency of parasite detection, exhibited low parasite burden (< 3 zoite/mg tissue) (**Figure 3A**). Comparative analysis of parasite burdens between infected groups showed higher loads in tissues from G2 compared to G1, with mean tissue burdens of 3.97 *vs*. 8.23 zoite/mg, respectively (p < 0.001, Mann−Whitney test). Analysis according to the tissue showed significantly higher parasite burden for G2 only in the *longissimus dorsi* muscle (p< 0.05; Mann−Whitney test) (**Figure 3F**). Although no significant differences were observed between parasite loads in the remaining tissues, G2 mean values were ten times higher in semimembranosus muscle, four times higher in brain, and almost three times higher in heart, tongue and diaphragm compared to G1 (**Figure 3**). The mean parasite burdens were approximately twice as high in the intercostal muscle from G1.

### Humoral immune response was higher with type III TgShSp24 isolate

**Figure 4:**
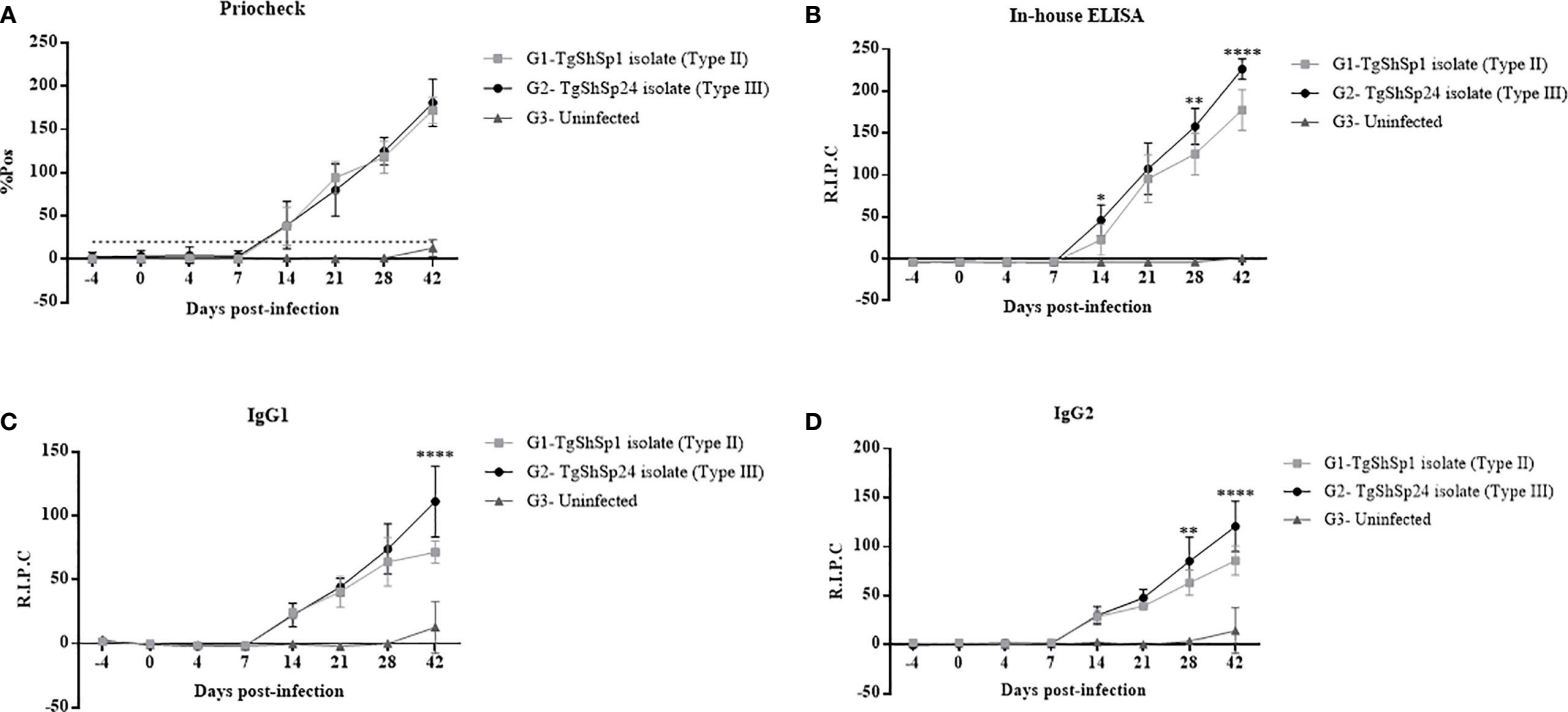
Evaluation of the IgG antibody levels by ELISA in sera from piglets infected with *T. gondii* oocysts from the TgShSp1 (G1) and TgShSp24 (G2) isolates and the non-infected group (G3). **(A)** Mean IgG levels by Priochek test are represented as the percentage of positivity (%PP). **(B)** Mean IgG levels by ELISA in house are represented as relative index percent (RIPC); **(C)** Mean RIPC IgG1 antibody levels; **(D)** Mean RIPC IgG2 antibody levels. Error bars represent SD. Significant differences between infected groups are indicated with asterisks, where *, ** and **** denote p < 0.05, p < 0.01 and p < 0.0001, respectively.

All animals from the infected groups (G1 and G2) displayed IgG seroconversion starting at 14 dpi by both ELISAs employed (p < 0.05; two-way repeated-measures ANOVA, Tukey’s post-test), and this IgG response progressively increased until 42 dpi. Animals from the uninfected group (G3) remained seronegative throughout the experiment. No significant differences were observed in IgG antibody levels between the infected groups as measured by the commercial test (PrioCHECK™) (**Figure 4A**). However, antibody levels were significantly higher in G2 than in G1 on 14 (p < 0.05), 28 (p < 0.01) and 42 dpi (p < 0.0001; two-way repeated-measures ANOVA, Tukey’s post-test) using the in-house ELISA (**Figure 4B**).

The IgG1 and IgG2 kinetic profiles were similar to those observed in the IgG response, also showing an increase in antibody levels after 14 dpi, with significant differences at 42 dpi (p < 0.0001) for IgG1 and 28 (p < 0.01) and 42 dpi (p < 0.0001; two-way ANOVA repeated-measures, Tukey’s post-test) for IgG2 between both infected groups (**Figures 4C, D**). Globally, piglets from G2 displayed significantly higher levels of IgG1 and IgG2 compared to those from G1. The IgG1/IgG2 ratio was not significantly different between the groups.

### Th1 cytokine profile was higher in piglets infected with type II TgShSp1 isolate

**Figure 5:**
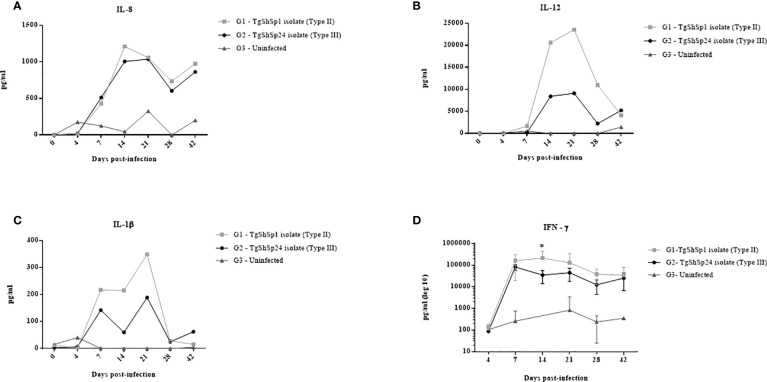
Cytokine levels measured in cell-free culture supernatants after specific *T. gondii* stimulation: IL-8 **(A)**, IL-12 **(B)** and IL-1β **(C)**. Each point represents the concentration of cytokines in the pooled supernatants for groups infected with TgShSp1 (G1), infected with TgShSp24 (G2) and uninfected control (G3) throughout the study. **(D)** IFN-ɤ responses measured in cell-free culture supernatants from individual animals of the G1, G2 and G3 groups. Each point represents the mean at the different sampling times for each group. Error bars represent SD. Significant differences between infected groups (*) indicate p < 0.05.

Analyses of cytokine secretion using the array after specific blood stimulation revealed a level increase (at least three times more the level in G3) in IL-8 and IL-1β from 7 dpi in both infected groups (G1 and G2) (**Figures 5A, B**). Cytokine levels of IL-12 were also increased from 7 dpi in G1 and from 14 dpi in G2. IL-8, IL-1β and IL-12 were maintained at high levels in infected groups G1 and G2 with respect to the control until the end of the experiment. IL-8 levels peaked at 14 dpi, without apparent substantial differences between the two infected groups (G1 and G2) (p > 0.05; Kruskal−Wallis and Dunn’s tests) (**Figure 5A**). For IL-1β and IL-12, the peak was reached at 21 dpi and then decreased significantly from 28 dpi onwards (**Figures 5B, C**). IL-1β and IL-12 levels were 1.9 and 2.6 times higher in G1 than in G2, respectively, at 21 dpi.

Individual data for 14 and 21 dpi on which the cytokine production peaks of IL-8, IL-1β and IL-12 were observed are shown in **Supplementary File 1**. Samples analysed individually exhibited a great dispersion of data among animals. Nevertheless, as the pooled results showed, IL-8 levels were high for infected groups G1 and G2, but only G2 displayed significant differences compared to the control group (G3) at both 14 and 21 dpi (p < 0.05; Kruskal−Wallis and Dunn’s tests). Mean IL-1β and IL-12 levels were also increased at day 21 dpi in G1 and G2 when they reached their peak as previously observed, although they only exhibited significant differences between G1 and G3 at 14 dpi (p < 0.01; Kruskal−Wallis and Dunn’s tests).

High levels of IFN-ɤ were also detected by array, although the standard curve failed to establish the linearity required for proper evaluation and was therefore disregarded (data not shown). Using a commercial kit (Porcine IFN-γ ELISA BASIC kit, Mabtech AB, Sweden), the production of IFN-ɤ was detected from 7 dpi in animals from both infected groups (G1 and G2) (**Figure 5D**). Significantly higher levels were observed in G1 than in G2 at 14 dpi (p < 0.05; two-way repeated-measures ANOVA, Tukey’s posttest).

GM-CSF, IL-4, IL-6, IL-10, TGF-β1, and TNF-α cytokine levels were not detected in either the infected groups or the uninfected group at any time throughout the experiment (data not shown).

## Discussion

Host-parasite interactions during *T. gondii* infection in livestock species such as pigs have not been sufficiently studied (4, 6–9). Here, we examined the interaction of *T. gondii* and piglets as well as the potential effect of two genotypes on the development of the disease (clinical outcome, immune response and parasite dissemination) and finally the persistence of the parasite in target tissues during the chronic phase. There is a growing body of evidence suggesting that the pathogenicity observed in a particular host species may not extrapolate to other hosts. In fact, a recent study found higher foetal mortality in sheep than in mice after infection with oocysts from the type-II TgShSp1 strain (20). Similarly, another report found no or low virulence in swine upon infection with the type III TgCatJpGil/TaJ isolate, as opposed to the high virulence observed in mice (26). When studying the TgCatJp4Ok4 isolate, which is phylogenetically close related to type II, virulence was high in both mouse and swine (26). This result again suggests that *T. gondii* differs in virulence depending on the host species. It is therefore not reliable to extrapolate the virulence data of each *T. gondii* strain from laboratory mice to domestic animals or humans (reviewed in 27).

Moreover, development of an experimental *in vivo* infection model is essential to understand the pathogenic mechanisms of *T. gondii* in swine with the potential to be extrapolated to humans. Pigs are a good animal model to study infection dynamics and associated immune responses due to their similarity to humans in terms of physiology, anatomy, dimensions, and immune responses (28, 29). This justifies the standardization of a porcine model of toxoplasmosis that will provide comparable parameters when animals are challenged with *T. gondii* isolates of different genetic backgrounds and phenotypic traits.

There are limited reliable studies that have led to the establishment of differences in virulence associated with the *T. gondii* genotype in pigs (8, 21). Numerous studies on experimental infections in pigs have been performed with diverse aims, such as the determination of the tropism and parasite load in different muscle groups, the development of diagnostic tools or the determination of vertical transmission; however, there is no standardization in the methods or parameters for comparison among studies and employed *T. gondii* isolates. Moreover, some studies were performed using the laboratory-adapted isolates ME49, RH or VEG [reviewed in (4)], and the maintenance history of some of these isolates is unknown; hence, the conclusions drawn in these studies may not reflect a natural infection scenario. A summary of these studies using reference laboratory isolates type I and II, RH and ME-49, respectively, revealed fever, apathy and anorexia of the animals during the acute phase (10-12 dpi), but all signs disappeared by 4 weeks pi, and the brain and heart were the most parasitized organs (4).

In the present study, two low-passage (not lab-adapted) isolates were used: TgShSp1 (Type II) and TgShSp24 (Type III), which possess genetic profiles representing those that circulate in swine (an in other hosts) in Europe (14). These two isolates of different genotypes exhibit low virulence and moderate-high capacity to form tissue cysts in mice. The two isolates were classified as nonvirulent in an outbred mouse model (15, 30). Specifically, a 0% cumulative mortality was observed for the type II TgShSp1 isolate and 20% for the type III TgShSp24 isolate, as well as a remarkably higher brain parasite load in the later, which suggests an enhanced dissemination ability of TgShSp24 in mice (19). On the other hand, the TgShSp1 isolate was described as the least virulent of those recently isolated in the Iberian Peninsula (19, 22). Moreover, since they have been recently isolated and maintained at low passage numbers in cell culture, it is reasonable to speculate that their phenotype in *in vivo* will mimic that of a natural infection, avoiding the impact of culture adaptation on phenotype (31).

Infection has been reproduced by inoculating different parasitic stages (tachyzoites, tissue cysts and oocysts) through different routes and in animals of different ages, which makes it difficult to compare the results obtained (7, 32–38). Previous experimental infections in pigs highlight the importance not only of the isolate but also of the dose to which animals are exposed and the parasitic stage in question [reviewed in (4)]. In another study, differences in antibody kinetics were observed when inoculating the same isolate (M4 strain - type II) at different stages, obtaining higher and earlier antibody levels in pigs infected with tissue cysts, in contrast to those infected with oocysts (34). Likewise, inoculating different doses of oocysts in pigs (10, 1 and <1) elicits decreasing antibody titres (32). Oocyst infections have mostly commonly been performed using 10^3^ – 5x10^3^ oocysts (4). In our work, we selected a dose of 10^3^ oocysts, since we aimed to recapitulate the mild presentation reported in natural infections, as well as the data on parasite dissemination in different tissues reported in the literature with this dose (36, 37).

The present experimental trial succeeded in mimicking the acute phase of the disease without severe clinical signs and subsequent development of chronic infection, as demonstrated by parasite detection in target tissues. Similar to previous observations in other studies where pigs were challenged with clonal and atypical *T. gondii* strains, animals developed fever and loss of appetite during the first 10 days, but later they fully recovered from the disease (4). In contrast, some experiments performed with YZ-1 isolate, Chinese 1 genotype (ToxoDB #9), (5x10^7^ tachyzoites intraperitoneally) or the closely related to type II Japanese TgCatJp4Ok4 (1x10^7^ tachyzoites intravenously) interestingly caused acute toxoplasmosis with severe clinical signs (respiratory distress, neurological signs and abortions), resulting in the death of some pigs (26, 39). However, another study that performed oral infection with 10^3^ oocysts of the PYS strain (ToxoDB #9) reported only fever and inappetence at 6 dpi (37). Therefore, the clinical manifestations observed may depend on the isolate, the dose or the parasite stage, in addition to parasite genotype. Our study demonstrated that the TgShSp24 (genotype #2, type III) isolate in piglets was accompanied by a more marked clinical response exhibiting higher temperatures and lasting longer. For all these reasons, the TgShSp24 isolate can be considered more virulent than TgShSp1 in piglets, which resembles the differences in virulence between these isolates that have been shown in mice (19).

Regarding parasite dissemination determined by mouse bioassay, the detection frequency was higher in tissues infected with the type III TgShSp24, which could be due to the higher load in these tissues and therefore the higher probability of infection, as well as the higher virulence of this isolate demonstrated in mice (likely by lower lethal dose fifty (LD50) than TgShSp1 not determined). Nevertheless, the pattern of parasite dissemination and burdens confirmed by qPCR demonstrate the higher dissemination and proliferation of TgShSp24 in pig tissues. A trend towards higher parasite burdens was also observed in another report where pigs were infected with a type II strain (*T. gondii* IPB-LR) compared to those infected with a hybrid type I/II strain (*T. gondii* IPB-Gangji) (8). As previously reported in the literature, a wide distribution of the parasite has been observed in tissues such as the brain and heart, as well as in other muscles of commercial interest (40–43). Similar tropism was observed for both TgShSp1 and TgShSp24 isolates in this study. In our experiment, tissues that presented the highest loads were the heart, tongue and masseter muscle, which agrees with previous studies (8, 40). However, contradictory results were also reported in another study in which loads in heart tissue were significantly lower than in the brain in animals challenged with 5x10^3^ oocysts of the type II strain (CZ-Tiger isolate) (7). In the present study, a histopathological study was not performed since according to the clinical signs observed during the trial and the results obtained with respect to the loads on the different tissues, which were not very high, it was not expected to provide additional relevant information.

*Toxoplasma gondii* infections in piglets were also revealed by IgG seroconversion with both isolate infections at two weeks post-inoculation, and IgG antibodies were gradually increased until the end of the experiment. Interestingly, antibody levels were higher in the group infected with the type III TgShSp24 isolate on all days except 21 dpi, which might be related to the type of immune response and/or the stimulus developed by the higher dissemination and parasite loads reached in piglet tissues. Similarly, seropositivity was observed in a study in which pigs were inoculated with 10^3^ oocysts of a type II (M4 strain) at day 14 pi, and IgG levels underwent a gradual increase between days 7 and 28 pi (34). Equally, in another work, pigs reached a serological positivity status at day 14 pi after inoculation of 5x10^3^ oocysts of a type II strain (CZ-Tiger isolate) (7). Seroconversion in most of the experimental studies usually occurs at 14 dpi with doses greater than 10^3^ oocysts. In contrast, that did not occur in a study in which all animals seroconverted at day 21 pi and were challenged with a type II strain (10^3^ oocyst) (36). The differences observed with respect to the days required to detect seroconversion after infection may be due to the inherent characteristics of the serological technique employed in each study. In the present work, two ELISA methods were used to confirm the infection of challenged piglets, and their implementation allowed us to observe statistically significant differences with the in-house ELISA between both groups. The differences found between the two tests could be due to the type of antigen, the secondary antibody used or the dilution of the sera.

The higher virulence observed in the group inoculated with the TgShSp24 isolate could be due to worse control by the developed immune response, as suggested by the cytokine response profile. Innate and cellular immune responses, *via* activation of CD4 and CD8 T lymphocytes, are relevant for the control of *T. gondii* infection in pigs (38). Activation of the immune system after *T. gondii* infection is dependent on factors such as cytoplasmic sensors (NLRP1) or caspases that produce the secretion of certain cytokines such as IL-1β and IL-18 in a process known as inflammasome activation. Moreover, in human peripheral blood mononuclear cells (PBMC), the secretion of these cytokines by the inflammasome is responsible for the secretion of IFN-ɤ and IL-12. The generation of IL-12 by dendritic cells (DCs) and macrophages is critical for host defence against toxoplasmosis, and it prepares NK and T cells (CD4 and CD8) to secrete IFN-ɤ (29, 44). In addition, cellular responses involving CD8 T cells and IFN-γ (45), along with innate immunity, have also been shown to lead to protection against *T. gondii* tissue cyst formation in pigs (45).

Cytokine analyses in this study revealed a predominance of a proinflammatory profile in response to both isolates in pigs. Production of the proinflammatory cytokines IL-1β, IL-12 and IFN-ɤ were detected together with the absence of the production of regulatory and anti-inflammatory cytokines such as IL-10 and IL-4 after specific stimulation in peripheral blood cells. In previous studies, similar results were observed determining the mRNA cytokine expression of proinflammatory cytokines in stimulated PBMCs in which the expression of the cytokines IL-10 and IL-4 was not detected, but IFN-ɤ was observed two weeks post-infection with 3x10^3^ tissue cysts of the type II IPB-G isolate (41, 42). Similarly, no expression of IL-10 or IFN-γ was observed one month after infection with 6x10^3^ tissue cysts of the type II IPB-G and type I/II IPB-LR isolates, but in contrast to our results, expression of IL-12 was not detected (21). Significant increases in the transcription of IFN-γ were also reported in the lymph nodes and spleen after infection with 10^3^ oocysts of the type II M4 isolate (38). All these studies highlight the Th1 profile response in cellular immune responses developed in pigs for the control of *T. gondii* infection. Clear clues in this study also suggest a stronger proinflammatory response induced by TgShSp1 oocyst infection than could explain the better control of the infection with this isolate in pigs. Cytokine IL-1β and IFN-γ levels were higher and IL-12 was detected earlier and higher with type II TgShSp1. In some studies, expression of the IL-12 costimulatory molecule CD40 was significantly increased in mice infected with type II *vs.* type I and III *T. gondii* parasites (46).

Earlier production of proinflammatory IL-8 was also induced by both TgShSp1-type II and TgShSp24-type III isolates in this study. IL-8 is a neutrophil chemoattractant that can be released by macrophages, DCs, as well as epithelial cells. Human intestinal epithelial cells infected with *T. gondii* elicit rapid secretion of IL-8 (47). IL-8 plays an important role in the innate immune response and inflammation, since neutrophils at the site of infection play a key role in the recruitment and activation of macrophages and DCs. IL-8 is considered the primary molecule of acute inflammation. IL-8 kinetics and its role on control of *T. gondii* infection has not been previously described in swine. Higher production of IL-8 by TgShSp24 infection was observed in this study, although apparently did not improved *T. gondii* control. New studies would be necessary to discern the role of IL-8 on the higher severity of clinical signs developed for acute infection by piglets infected with TgShSp24 isolate.

*Toxoplasma gondii* isolates also differ in their ability to induce an immune response. Differences in virulence between strains are directly related to polymorphic factors that modulate the immune response (29). All strains actively express the *GRA24* effector; however, type II strains induce a much stronger proinflammatory response than type I or III strains due to the expression of *GRA15* and the absence of active *ROP16*. *GRA15* type II has been found to determine *T. gondii* strain differences in modulating the inflammatory response. Together, *GRA15* and *GRA24* drive the classical activation of macrophages (M1) *via* the activation of NFκB and p38 MAPK, inducing the production of inflammatory cytokines such as IL-12 and IL-1β with an improved efficiency in eliminating intracellular pathogens in mammals lacking TLR11 (29, 48). The findings in this study also suggest a stronger Th1 proinflammatory response for TgShSp1-type II infection. On the other hand, *ROP16* (absent in type II) induces alternative activation of macrophages (M2), which are less efficient at eliminating intracellular pathogens and are associated with an increased Th2 response. Of note, in type II strains, parasite clearance may take place earlier due to the high proinflammatory response, but in some cases, this may also cause disease due to an excessive induction of the immune response in the host that can lead to ileitis or encephalitis (29). These effectors should be studied further, not only in different models than the mouse but also using newly obtained isolates.

In our study, both isolates elicited a proinflammatory Th1 profile in pigs, although the levels were higher with the TgShSp1 isolate, also coinciding with lower levels of humoral immunity. This immune response profile could be an important factor influencing the persistence of the parasite and the higher burdens observed in the tissues of animals infected with the TgShSp24 strain, which had higher antibody levels but lower cellular response. Therefore, differences observed throughout the study could be influenced and partially explained by parasite-dependent factors, such as strain-specific effectors or the specific phenotype involving multiplication, dissemination or immune response avoidance, among others. On the other hand, the response elicited by the immune system of the host against the parasite also surely plays an important role in limiting the infection.

In summary, in the present study, we describe a model that allows the characterization and comparison of different isolates in piglets. Similar to other animal models, parasite genotype appears to influence the immune response and the final establishment of chronic infection in pigs. TgShSp24 (type III) was more virulent than TgShSp1 (type II), as observed by clinical data, dissemination and parasite loads in different tissues. Moreover, these findings indicate that a Th1 response induced at higher levels by the archetypal type II strain may be related to improved control of the infection of the TgShSp1 isolate, associated with lower parasite multiplication and dissemination in pig tissues. Additional studies using the current normalized pig model are warranted to test the efficacy of potential vaccines and therapeutic molecules against chronic infection by type II and type III isolates in pigs.

## Data availability statement

The original contributions presented in the study are included in the article/**Supplementary Material**. Further inquiries can be directed to the corresponding authors.

## Ethics statement

Animal procedures were approved by the Animal Welfare Committee of the Community of Madrid, Spain (PROEX 293.7/20, PROEX 290.4/20 and PROEX 062/19), following procedures described in Spanish and EU regulations (Law 3/2007, R.D. 53/2013, and Council Directive 2010/63/EU). All animals (piglets, mice and cats) used in this study were handled in strict accordance with good animal practices, and all efforts were made to minimize their suffering.

## Author contributions

LMO-M, JR-C, IF, RC-B, CD-D, and RS-S conceived the study and participated in its design. AL, CD-D, GA-C, and RS-S participated in inoculation and clinical examination of animals, performed necropsies and sampling of the animals. RS-S carried out the oocyst production. AL, CD-D, and JR-C analysed the data, carried out statistical analyses and interpreted the results. AL and JR-C wrote the manuscript with result interpretation and inputs from CD-D, RC-B, RS-S, IF, and LMO-M. All authors contributed to manuscript revision, read, and approved the submitted version.

## Funding

CDD was finantially supported by by the Spanish Ministry of Science and Innovation and the European Social Fund (PTQ2019-010719) and ALT by the Spanish Ministry of Science and Innovation and the European Union “NextGenerationEU/PRTR” (DIN2020-011454/AEI/10.13039/501100011033).

## Acknowledgments

We gratefully acknowledge Silvia Jara Herrera for her excellent technical assistance and Juan José de Andrés Cercas for his help during the animal work. We also thank Dr. David Arranz and Prof. Gema Álvarez for their critical comments to this manuscript.

## Conflict of interest

The authors declare that the research was conducted in the absence of any commercial or financial relationships that could be construed as a potential conflict of interest.

## Publisher’s note

All claims expressed in this article are solely those of the authors and do not necessarily represent those of their affiliated organizations, or those of the publisher, the editors and the reviewers. Any product that may be evaluated in this article, or claim that may be made by its manufacturer, is not guaranteed or endorsed by the publisher.

## Supplementary material

Supplementary File 1 Cytokine levels measured in cell-free culture supernatants after specific *T. gondii* stimulation at 14 and 21 days post-infection (dpi): IL-8 **(A, B)**, IL-12 **(C, D)** and IL-1β **(E, F)**. Box-plot graphs representing median and 25^th^ to 75^th^ percentiles of cytokine levels of the individual values from groups infected with TgShSp1 (G1), TgShSp24 (G2) and non-infected (G3). Whiskers represent the largest and smallest values Significant differences between groups are indicated with asterisks, where * and ** indicates p < 0.05 and p < 0.01, respectively. δ denotes a trend towards significance p < 0.1.
