# Supplementary figures and images for: Archetypal type II and III Toxoplasma gondii oocysts induce different immune responses and clinical outcomes in experimentally infected piglets
Source: Front Immunol. 2022 Oct 20;13:1021556. doi: 10.3389/fimmu.2022.1021556 (PMC9631316; doi:10.3389/fimmu.2022.1021556)

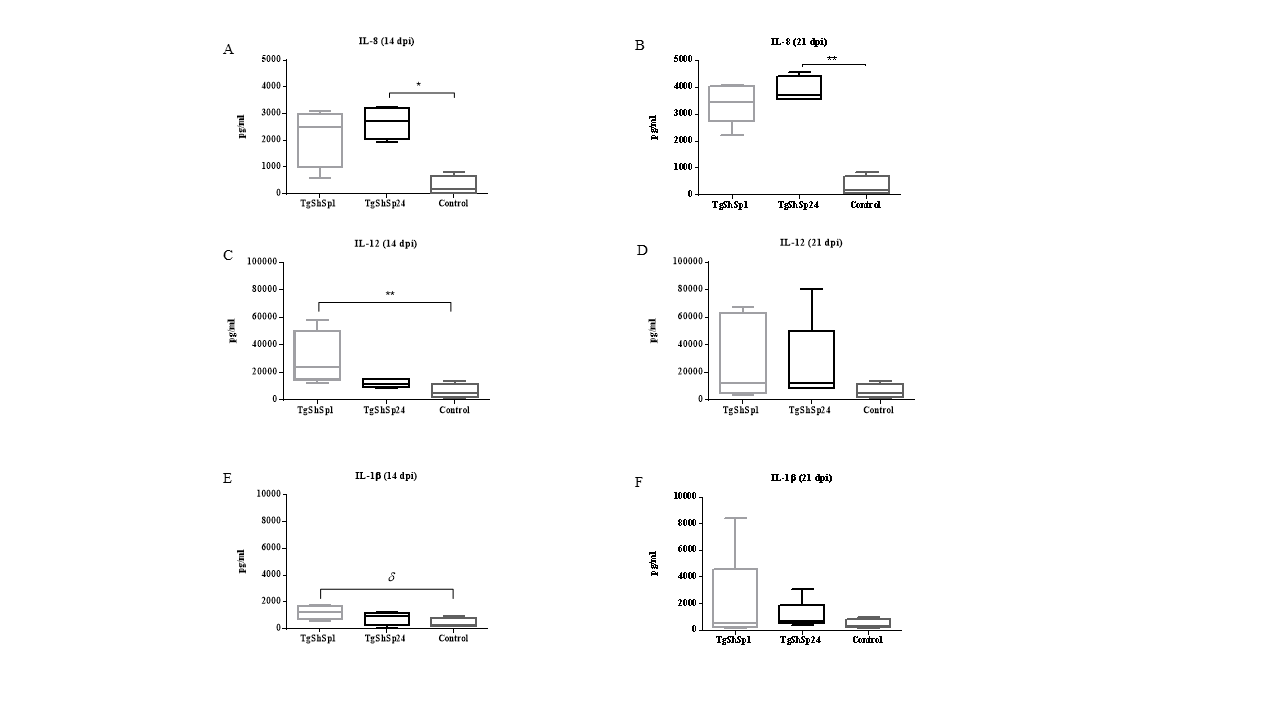

Supplement: Supplementary File 1 — Cytokine levels measured in cell-free culture supernatants after specific T. gondii stimulation at 14 and 21 days post-infection (dpi): IL-8 (A, B), IL-12 (C, D) and IL-1β (E, F). Box-plot graphs representing median and 25th to 75th percentiles of cytokine levels of the individual values from groups infected with TgShSp1 (G1), TgShSp24 (G2) and non-infected (G3). Whiskers represent the largest and smallest values Significant differences between groups are indicated with asterisks, where * and ** indicates p < 0.05 and p < 0.01, respectively. δ denotes a trend towards significance p < 0.1. [file Image_1.tif]
